# The identification of family subtype based on the assessment of subclinical levels of psychosis in relatives

**DOI:** 10.1186/1471-244X-12-71

**Published:** 2012-07-03

**Authors:** Eske M Derks, Marco PM Boks, Jeroen K Vermunt

**Affiliations:** 1 University Medical Center Utrecht, Department of Psychiatry, Rudolf Magnus Institute of Neuroscience, AMC-APC, room PA1-179 Meibergdreef, Amsterdam, AZ 51105, The Netherlands; 2 Department of Methodology and Statistics, Tilburg University, Tilburg, The Netherlands; 3 Department of Psychiatry, Academic Medical Centre University of Amsterdam, Amsterdam, The Netherlands

**Keywords:** Family subtype, Familial loading, Multiplex, Sporadic, Phenotypic heterogeneity, Schizophrenia, Controls, Factor analysis, Latent class analysis, Mixed model latent class analysis

## Abstract

**Background:**

Schizophrenia is a complex psychiatric disorder characterized by high phenotypic heterogeneity. Previous studies have distinguished between familial and sporadic forms of schizophrenia and have suggested clinical differentiation between patients and relatives from sporadic and multiplex families. We will introduce a more refined method to distinguish between family subtypes based on psychosis dimension profiles in the relatives of schizophrenia patients.

**Methods:**

Positive, negative, disorganization, mania, and depression scores were assessed in 1,392 relatives. Mixed Model Latent Class Analysis was used to identify family subtypes. A family subtype is a relatively homogeneous group of families with similar symptom profiles in the relatives in these families. Next, we investigated in 616 schizophrenia patients whether family subtype was associated with symptom profiles, IQ, cannabis dependence/abuse, or age of onset of psychosis.

**Results:**

Based on the data of relatives, we identified two different family types: “healthy” and “at risk for psychiatric disorder”. Patients from at risk families obtained higher positive scores compared to patients from healthy families (Wald(1) = 6.6293, p = 0.010). No significant differences were found in any of the remaining variables.

**Conclusions:**

Our findings confirm the existence of high-risk families and although we did not establish an etiological basis for the distinction between family types, genetic studies might reveal whether family subtype is associated with genetic heterogeneity.

## Background

Schizophrenia is a complex psychiatric disorder characterized by high phenotypic heterogeneity within patients [1-3]. This phenotypic heterogeneity has been studied using factor analysis (i.e., variation in symptoms being explained by continuous latent factors) and latent class analysis (i.e., variation in symptoms being explained by the presence of different clusters of patients) or a combination of these approaches [3,4]. We previously reported on the existence of five latent dimensions (i.e., disorganization, positive, negative, mania, and depression) and seven different classes of subjects in a large sample (N > 4,000) of schizophrenia patients, their relatives and healthy controls. The classes and dimensions were validated by showing associations with IQ and daily functioning [3], and progressive volume changes in brain volumes in schizophrenia patients [5]. In the present study, we will extend these previous analyses by incorporating heterogeneity at the family level using a relatively new statistical approach: mixed model Latent Class Analysis. We aim to distinguish between different family subtypes based on psychosis dimension scores assessed in relatives. As dimensional scores provide a more refined measure compared to diagnosis, our study may facilitate the investigation of the etiological causes of schizophrenia by improving the distinction between familial and sporadic forms of schizophrenia.

Previous studies have distinguished between familial and sporadic forms of schizophrenia by investigating differences between multiplex (i.e., pedigrees with multiple affected individuals) and sporadic (i.e., families with a single affected individual) families. Even though schizophrenia is a highly heritable disorder [6,7], the majority of the schizophrenia patients are sporadic cases, which is expected under the genetic liability model as shown by Yang and colleagues [8]. It is yet unclear whether multiplex and sporadic families are characterized by different etiological factors. Studies comparing sporadic versus multiplex families have revealed a relatively low age of onset [9], poor outcome [9,10] and more impairment of working memory [11] in patients from multiplex families. However, sporadic cases showed more alcohol abuse [12]. Likewise, non-psychotic siblings of patients in multiplex families showed decreased cognitive functioning including impaired sustained attention [13], worse performance on visual working memory tasks [14] and more executive deficits [15]. Whether these clinical differences are representative of heterogeneity in the underlying etiology needs further investigation.

Unfortunately, previous studies are hindered by an oversimplified distinction between sporadic and multiplex families as some of the relatives of patients with schizophrenia may not meet the criteria for a psychiatric diagnosis but may suffer from subclinical psychosis symptoms [16]. Family members of schizophrenia patients may be more prone to show a broad range of subclinical symptoms compared to the general population; these symptoms may therefore be important to identify family subtypes. We will use multilevel Latent Class analysis (LC) developed by Asparouhov and Muthen [17], and Vermunt [18] as this approach allows for the identification of classes of families with similar patterns of symptom dimension scores in subjects belonging to these families.

In this paper we will address two questions. First, we will investigate whether different subtypes of families can be distinguished based on psychosis dimension scores in relatives of schizophrenia patients. Second, we will investigate whether there is an association between family subtype and risk factors for schizophrenia (e.g., cannabis use), or patient characteristics (e.g., IQ, psychosis dimension scores).

## Methods

### Subjects

Subjects were recruited as part of the Genetic Risk and Outcome of Psychosis (GROUP). In the GROUP study, patients were identified in selected representative geographical areas in the Netherlands and Belgium. All patients met the following criteria: (1) age between 16 and 50, (2) Diagnostic and Statistical Manual of Mental Disorders, Fourth Edition (DSM-IV) criteria for a psychotic disorder (including schizophrenia, schizophreniform disorder, and schizoaffective disorder), (3) fluency in Dutch, and (4) written informed consent. Eligible siblings of schizophrenia patients (brothers and/or sisters) fulfil the criteria of (1) age between 18 and 50 (extremes included), (2) fluency in Dutch, and (3) written informed consent; except for the age criterion, similar criteria applied to the parents. The GROUP study was approved by the Medical Ethics Committee (METC) of UMC Utrecht, the Netherlands and all subjects gave written informed consent in accordance with the committee’s guidelines.

In this study we included data of families with at least one participating relative. In one of the four participating centers, DSM-IV diagnosis was assessed with the Schedules for Clinical Assessment in Neuropsychiatry (SCAN) instead of the Comprehensive Assessment of Symptoms and History (CASH) and patients recruited in this center were excluded from analyses due to incompatibility of the symptom assessment. The resulting sample included 1,392 relatives from 671 families. Patient data were available in 616 of these families.

### Measures

#### CASH symptom dimensions

All subjects were assessed with the Comprehensive Assessment of Symptoms and History (CASH) interview [19]. Interviews were administered by research assistants (primarily psychologists and psychiatrists) who attended structured training workshops. Assessments were supervised by WC, LdH, RB, IM-G, or LK for the GROUP study, and by WC or RH for the Utrecht study. A subset of 63 items was used to estimate factor scores of five underlying dimensions: positive, negative, depression, mania, and disorganisation [3]. Due to the non-normality of the distribution of the factor scores, we recoded the continuous scores and created an ordinal three-point scale.

#### Cannabis

Based on the lifetime rated Cannabis abuse subscale of the Composite International Diagnostic Interview (CIDI; [20]), a distinction was made between four groups of subjects: no dependence or abuse, dependence, abuse, and dependence and abuse. Cannabis dependence/abuse was rated in all 616 patients.

#### WAIS-IQ

All subjects were assessed with a comprehensive neurocognitive test battery containing the following tasks (intended cognitive domains of focus are placed between brackets): WAIS-III Digit Symbol–Coding (processing speed) (15), Continuous Performance Test-HQ (attention/vigilance) (16, 17), Word Learning Task (verbal learning and memory) (18), WAIS-III Arithmetic (working memory) (15), WAIS-III Block Design (reasoning and problem solving) (15), Response Shifting Task (set-shifting), which is a modified version of the Competing Programs Task (19, 20), and WAIS-III Information (verbal comprehension) (15). To calculate a measure of global cognitive functioning (i.e., IQ), raw test scores were converted into standardized z-scores against the means and standard deviations of the healthy control group. Z-scores were calculated for each cognitive domain and were recoded if necessary such that more negative z-scores reflected worse performance for all measures. IQ was assessed in 604 (98%) of the patients.

#### Camberwell assessment of need

The Camberwell Assessment of Need (CAN) rating scale [21] was used to assess met and unmet clinical and social needs in the patients participating in the GROUP study. The CAN rating scale consists of 22 items rated on a 0–2 scale. The CAN was assessed in 582 (95%) of the patients.

#### Age of onset first psychosis

The age of onset of the first psychotic symptoms was assessed based on the CASH interview and was completed by 614 (99.7%) of the patients.

### Statistical analyses

For a formal introduction to the multilevel LC model, we refer to Vermunt [18]. We used the non-parametric variant of the multilevel LC model. In short, in this model differences across groups (i.e., families) are modeled using a discrete latent variable at the group level. There are three levels present in this analysis. First, there are the observed responses (e.g., symptom ratings) of an individual at the lowest level. Next, individuals may be clustered using a lower level LC, this step corresponds to a conventional LC analysis (e.g., identification of symptom profiles). The lower level latent classes are assumed to differ in the distribution of the observed responses. At the third and highest level, the family structure of the data is taken into account. The higher level LCs differ in the distribution of the lower-level LCs and group families into different family types. When selecting the best-fitting model, a decision has to be made both on the number of lower-level and of higher-level LCs. We followed the guidelines proposed by Lukociene et al. [22] and performed a three-step procedure. First, we determined the number of lower-level classes ignoring the multilevel (family) structure of the data. Second, we fixed the number of lower-level classes to the value of step 1 and determined the number of higher-level classes. Third, we fixed the number of higher-level classes to the value of step 2 and redetermined the number of lower-level classes. Model fit of nested models was compared based on the Bayesian Information Criterion (BIC) [23] which is the preferred criterion for simultaneously deciding about the number of lower- and higher-level classes [22]. The BIC was calculated using the number of families as the number of observations. Statistical analyses were performed in LatentGold [24] scripts will be made available at request).

After establishing the presence of distinctive family subtypes, we compared variables of interest between family subtypes. To adjust for possible classification errors occurring when assigning families to their most likely (modal) class, in these post-hoc analyses, we used the modified BCH adjustment as described by Vermunt [25]. This approach involves constructing an expanded data set with the inverse of the classification error matrix as weights. Parameter estimation in subsequent analyses involves maximizing a weighted log-likelihood for clustered data, which means that complex sampling variance estimation methods need to be used to obtain correct statistical tests. First, we tested which specific symptoms were scored significantly different by the relatives of the different family subtypes. Next, we investigated the validity of family subtypes by testing whether CASH symptom dimension scores in patients were significantly different between family subtypes. The type-I error rate was Bonferroni corrected for the five tests that were performed and was set at .05/5 = .01. We also investigated whether the etiological measures: WAIS-IQ, CAN met and unmet needs, Cannabis abuse/dependence, and age of onset first psychosis differed significantly between family subtypes. Again, the type-I error rate was set at .01.

## Results

### Mixed model latent class analyses

**Figure 1:**
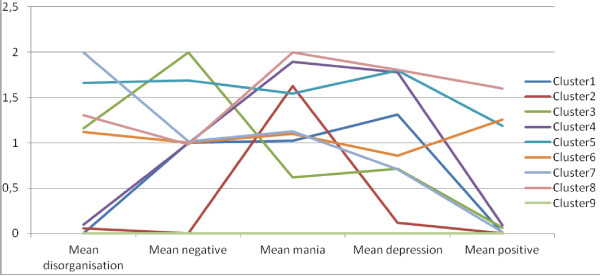
Mean dimension scores by latent class.

**Figure 2:**
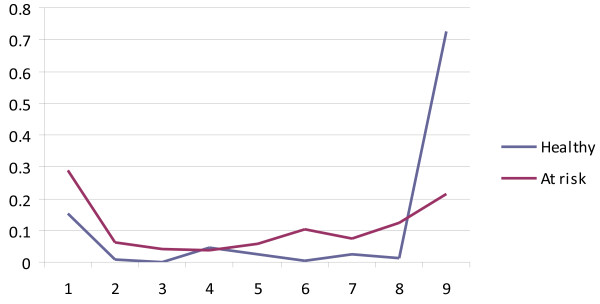
Latent class probability by family type.

As mentioned in the method Statistical Analyses section, we used a three-step procedure to decide on the number of lower-level and higher-level latent classes (see Table 1). At step 1, we included only one higher-level class and increased the number of lower-level latent classes. According to the BIC, a model with nine lower-level latent classes provided the best fit. Next, we tested whether the model fit improved if the number of higher-level classes was increased. Our analyses showed that a model with 2 higher-level latent classes provided a better fit compared to a model with one or three higher-level latent classes. Finally, we tested whether the optimal number of lower-level latent classes changed due to the inclusion of two higher-level latent classes. The number of lower-level latent classes did not change: our final model included nine lower-level latent classes and two higher level classes. In other words, individual subjects were assigned to nine different classes while we identified two different types of families. The 2 group-9 class model is visualized in Figures 1 and 2. Figure 1 shows the mean dimension scores in the nine individual-level latent classes. Figure 2 represents the latent class probabilities in the two family subtypes. Slightly more families were identified as “at risk for psychiatric disorder” families (57%) than “healthy” (43%). While subjects assigned to the “healthy” family type have a high chance (73%) of being assigned to the no-symptoms class (Cluster 9), subjects from "at risk for psychiatric disorder" families have a 20% chance of being assigned to the no-symptoms class while the remaining 80% is assigned to any of the other classes (see Figure 2). Table 2 shows the demographic data in the relatives in the total sample and by family type.

**Table 1:** Comparison of model fit in the Mixed Model LCA analyses

	**Npar**	**BIC**
**STEP 1, determine the number of lower-level latent classes**		
Model 1. 1 higher-level latent class, 1 lower-level latent class	10	11662.99
Model 2. 1 higher-level latent class, 2 lower-level latent classes	16	8339.47
Model 3. 1 higher-level latent class, 3 lower-level latent classes	22	7749.42
Model 4. 1 higher-level latent class, 4 lower-level latent classes	28	7560.36
Model 5. 1 higher-level latent class, 5 lower-level latent classes	34	7420.44
Model 6. 1 higher-level latent class, 6 lower-level latent classes	40	7368.64
Model 7. 1 higher-level latent class, 7 lower-level latent classes	46	7307.69
Model 8. 1 higher-level latent class, 8 lower-level latent classes	52	7298.12
**Model 9. 1 higher-level latent class, 9 lower-level latent classes**	**58**	**7308.68**
Model 10. 1 higher-level latent class, 10 lower-level latent classes	64	7330.06
**STEP 2, determine the number of higher-level latent classes**		
Model 11. 1 higher-level latent class, 9 lower-level latent classes (=model 9)	58	7308.68
**Model 12. 2 higher-level latent classes, 9 lower-level latent classes**	**67**	**7261.36**
Model 13. 3 higher-level latent classes, 9 lower-level latent classes	76	7297.57
**STEP 3, redetermine the number of lower-level latent classes**		
Model 14. 2 higher-level latent classes, 8 lower-level latent classes	60	7268.99
**Model 15. 2 higher-level latent classes, 9 lower-level latent classes (=model 12)**	**67**	**7261.36**
Model 16. 2 higher-level latent classes, 10 lower-level latent classes	74	7272.55

Note: the best-fitting models in each of the steps are shown in bold.

**Table 2:** Overview of demographic and clinical data in relatives in the total sample and by family subtype

		**Total sample (N = 1392)**	**“Healthy” (N = 637)**	**“At risk” (N = 755)**	**Test statistic (df)**	**P**
Mean age (SD)		40.37 (15.68)	40.40 (15.41)	40.35 (15.91)	Wald(1) = .00	.95
N male (%)		605 (43.5)	280 (44.0)	325 (43.0)	Wald(1) = .12	.73
N diagnosis (%)	Control	1111 (79.8)	586 (92)	525 (69.5)	Wald(2) = 95.93	<.001
	Depression	258 (18.5)	42 (6.6)	216 (28.6)		
	Rest	23 (1.7)	9 (1.4)	14 (1.8)		
WAIS-IQ		103.65 (16.15)	104.11 (15.72)	103.28 (15.91)	Wald(1) = 0.90	.34

Note: - As a posthoc analysis, we repeated these analyses in relatives unaffected for depression. The differences between relatives from the two subtypes became somewhat smaller, but were still highly significant.

- These analyses were performed in LatentGold and were corrected for family structure by taking into account possible correlations within families.

We have performed a simulation study to investigate the statistical power to differentiate between the 1 group-9 class model and the 2 group-9 class model. Data were simulated with the estimates of the best fitting model as parameter values; 500 replicates were performed. The fit of the two models was compared based on the BIC. The results of this simulation study showed that the 2 group-9 class model provided a better fit compared to the 1 group-9 class model in 492 of the 500 replicates (98%) which shows that the statistical power to differentiate between these models is excellent.

### A more detailed Investigation of the family subtypes

The two family subtypes are distinguished based on the latent class probabilities of individuals belonging to a particular family. The individual latent classes are characterized by a particular profile on the five psychosis dimensions. The data reported in Table 2 show that all five symptom dimension scores were significantly higher in “at risk for psychiatric disorder” compared to “healthy” families (all p < .001). As the prevalence of depression is also higher in relatives from “at risk for psychiatric disorder” families (28.6% compared to 6.6% in healthy families), we investigated whether the higher depression rate explained the different psychosis dimension scores, by repeating the analysis in relatives unaffected for depression. The differences between relatives from the two subtypes became somewhat smaller, but were still highly significant.

**Figure 3:**
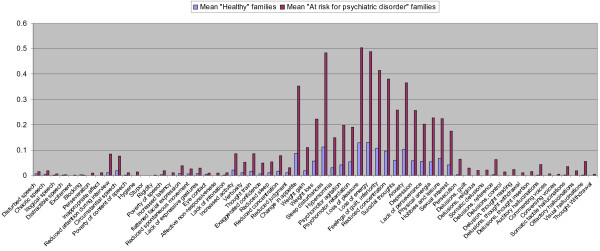
Mean scores on specific symptoms in the relatives of “healthy” and “at risk for psychiatric disorder” families.

We next explored whether particular symptoms are responsible for the distinction between family subtypes. To this end, we compared the 63 symptoms directly between the “at risk for psychiatric disorder” and “healthy” families. The mean scores of the two family types are shown in Figure 3. We performed one-sided statistical tests to investigate which symptoms are more prevalent in “at risk for psychiatric disorder” families compared to “healthy” families at a type-I error rate of .05/63 = .00079. The majority of the positive (15/16 symptoms, 94%), depression symptoms (18/18 symptoms, 100%), and mania symptoms (6/7 symptoms, 86%) were more prevalent in “at risk for psychiatric disorder” families compared to “healthy” families”. In contrast, few of the negative (1/10 symptoms, 10%) or disorganization (5/12 symptoms, 42%) were significantly different between family subtypes.

We also explored whether differences in symptom dimension scores between family subtypes were present only in siblings or parents. The results show that differences are similar in these two types of relatives (see Table 3).

**Table 3:** Symptom dimension scores in relatives by family type

		**Total sample (N = 1392)**	**“Healthy” (N = 637)**	**“At risk” (N = 755)**	**Test statistic (df)**	**P**
All relatives						
CASH symptom dimensions	Disorganization	-.21 (.32)	-.35 (.19)	-.09 (.35)	Wald (1) = 331	<.001
	Negative	-.21 (.29)	-.36 (.18)	-.08 (.29)	Wald (1) = 500	<.001
	Mania	-.26 (.44)	-.49 (.31)	-.07 (.45)	Wald (1) = 471	<.001
	Depression	-.31 (.58)	-.65 (.44)	-.03 (.53)	Wald (1) = 631	<.001
	Positive	-.46 (.35)	-.68 (.19)	-.28 (.34)	Wald (1) = 776	<.001
Parents		N = 647	N = 289	N = 358		
CASH symptom dimensions	Disorganization	-.19 (.34)	-.32 (.25)	-.09 (.37	Wald (1) = 84	<.001
	Negative	-.18 (.30)	-.33 (.25)	-.07 (.30)	Wald (1) = 139	<.001
	Mania	-.27 (.39)	-.45 (.35)	-.13 (.37)	Wald (1) = 125	<.001
	Depression	-.26 (.59)	-.56 (.53)	-.02 (.53)	Wald (1) = 169	<.001
	Positive	-.46 (.31)	-.64 (.22)	-.31 (.30)	Wald (1) = 258	<.001
Siblings		N = 745	N = 348	N = 397		
CASH symptom dimensions	Disorganization	-.23 (.29)	-.38 (.10)	-.09 (.33)	Wald (1) = 272	<.001
	Negative	-.23 (.27)	-.40 (.12)	-.08 (.28)	Wald (1) = 420	<.001
	Mania	-.25 (.48)	-.52 (.27)	-.01 (.49)	Wald (1) = 316	<.001
	Depression	-.35 (.57)	-.72 (.34)	-.03 (.53)	Wald (1) = 442	<.001
	Positive	-.46 (.37)	-.71 (.15)	-.24 (.37)	Wald (1) = 539	<.001

### A comparison of patients from “at risk for psychiatric disorder” and “healthy” families

#### Clinical characteristics

Lifetime assessed psychosis dimension scores indicated higher positive symptom scores in patients from “at risk for psychiatric disorder” families compared to patients from “healthy” families (Wald(1) = 6.6293, p = 0.010). There were no apparent differences in the other symptom domains, including negative, disorganization, mania, general, and depression symptoms (all p > 0.10). There were also no significant differences in the remaining clinical characteristics, including WAIS-IQ, or the number of met or unmet needs. The results of these analyses are summarized in Table 4.

**Table 4:** A comparison of family and patient characteristics by family type

		**Healthy (N = 272)**	**At risk (N = 344)**	**Test statistic (df)**	**P**
**Patient characteristics (demographic)**					
Mean age (SD)		27.8 (7.7)	27.0 (7.5)	Wald (1) = 1.43	.23
N male (%)		217 (79.8)	255 (74.1)	Wald (1) = 2.69	.10
**Patient characteristics (clinical)**					
CASH symptom dimensions	Disorganization	.56 (.48)	.57 (.46)	Wald (1) = .15	.70
	Negative	.58 (.50)	.59 (.47)	Wald (1) = .03	.87
	Mania	.58 (.57)	.60 (.55)	Wald (1) = .20	.66
	Depression	.39 (.44)	.43 (.41)	Wald (1) = 1.09	.30
	Positive	.74 (.46)	.84 (.45)	Wald (1) = 6.63	.01
Mean WAIS-IQ (SD)		94.8 (15.24)	94.8 (15.66)	Wald (1) = .00	.97
CAN	Met needs	3.26 (2.95)	3.21 (3.89)	Wald (1) = .02	.89
	Unmet needs	4.05 (2.92)	4.08 (3.07)	Wald (1) = .04	.85
**Patient characteristics (etiological)**					
Number of cannabis users (%)	No abuse/dependence	159 (58.5)	198 (57.6)	Wald (3) = 7.94	.05
	dependence	77 (28.3)	75 (21.8)		
	abuse	25 (9.2)	43 (12.5)		
	dependence and abuse	11 (4.0)	28 (8.1)		
Age of onset first psychosis		23.0 (7.2)	22.1 (6.7)	Wald (1) = 2.72)	.10

#### Etiological factors

The hypothesized etiological factors (i.e., cannabis (ab)use or dependence and age of onset) were not significantly different between patients from the two different family types (see Table 4).

## Discussion

Using psychosis dimension scores assessed in a sample 1,392 relatives of schizophrenia patients from 671 families we identified two family subtypes: “at risk for psychiatric disorder” and “healthy”. These family subtypes were mainly distinguished based on depression, mania, and positive symptoms. We subsequently investigated whether patient characteristics were different between family types. A difference was revealed in the level of positive symptoms; patients from “at risk for psychiatric disorder” families obtained higher positive symptom scores compared to patients from “healthy” families.

Previous studies have distinguished between sporadic and multiplex families based on either a simple distinction between families with one or more schizophrenia patients, or using a more sophisticated algorithm which takes the number of family members, the degree of genetic relatedness, age at the time of the study and gender into account [26]. Differences in clinical presentation of patients from sporadic and multiplex families have been reported, but results were inconsistent; it remained unclear whether clinical differences represented etiological heterogeneity. In the present study, we used a relatively new statistical approach to perform a family based analysis of the subclinical levels of psychosis in family members of schizophrenia patients. We identified two different family types: 42% of the families was assigned to the “healthy” family class while 58% of the families was assigned to the “at risk for psychiatric disorder” family class. Relatives assigned to the “at risk for psychiatric disorder” were more often diagnosed with depression and obtained higher scores on the five psychosis dimensions (i.e., positive, negative, depression, mania, and disorganization) compared to relatives from “healthy families”. The higher psychosis dimension scores were not solely due to the higher prevalence of depression, as the differences between relatives were slightly less pronounced but still significantly different in relatives unaffected for depression (data not shown).

The main novelty of the approach presented in this article is the use of refined phenotypes to define family subtype. The distinction between family types is based on symptom dimension scores of all participating family members instead of a dichotomous distinction between affected and unaffected. Although our approach is much more labor-intensive in terms of data collection (i.e., the collection of symptoms instead of psychosis yes/no) the extra information appears to contribute to a valid assessment of family type.

Patients from the “at risk for psychiatric disorder” family type obtained higher scores on lifetime rated positive symptoms (i.e., the CASH positive dimension) compared to patients from the healthy family type. The difference was specific for positive symptoms; no differences were found for the remaining symptom dimensions, IQ, the number of needs, Cannabis abuse/dependence or age of onset of psychosis. This is in contrast to the scores in relatives while parents and siblings of the different family subtypes obtain different symptom scores on all five psychosis dimensions, with the largest differences found for positive, depression, and mania dimensions.

We will discuss two competing explanations for our findings which indicate that positive symptoms in patients are most strongly associated with positive, depression, and mania scores in their relatives and not with negative and disorganization symptoms. The first argument will be built on the premise that schizophrenia results from an increased risk for cognitive deterioration and an increased risk for psychotic features. We have previously shown the existence of a subset of schizophrenia patients with relatively low disorganization and negative symptoms and normal cognitive functioning [3]. This suggests that disorganization and negative symptoms may be indicators, or clinical correlates, of the cognitive decline in schizophrenia patients. The fact that the scores on disorganization and negative symptoms in the “at risk for psychiatric disorder” relatives are relatively unaffected may suggest that these relatives do share the risk for developing psychotic features with their affected family member while they do not share the risk for cognitive deterioration. This would imply that negative symptoms, disorganization and cognitive decline are etiologically distinct from the other symptom clusters.

A second explanation is based on the argument that the relatively high level of positive symptoms in patients with schizophrenia in “at risk” families may not result from the increased familial loading for psychosis but may instead be related to the increased psychosis dimension scores in their relatives via a causal pathway whereby higher positive scores in siblings or offspring result in increased distress and lead to higher scores as a reflection of familial distress due to a more severe presentation of the illness. Such distress is more likely to be reflected in mania, positive, and depression scores than in disorganization and negative symptoms.

Cannabis abuse and/or dependence was not present at significantly different rates in patients from “at risk for psychiatric disorder” and “healthy” families. In addition, age of onset of psychosis was not significantly different between groups. Does this imply that our novel classification for family types does not have an etiological basis? Not necessarily, since the most important etiological risk factor: genetic variation, was not yet taken into account. Future studies should reveal whether genetic risk for schizophrenia, for example such as calculated in the study by Purcell and colleagues [27], may be significantly different between subjects from “at risk for psychiatric disorder” and “healthy” families. We hypothesize that relatives from “at risk” families have a higher mean genetic risk score compared to relatives from “healthy” families, even though the relatives in both family types do not cross the threshold of “being affected”. If our symptom based differentiation between “at risk” and “healthy” families will be confirmed by genetic studies, this will have important implications for future gene finding studies. The statistical power to detect genetic risk factors involved in schizophrenia could be increased by selecting those families with a high genetic loading.

The results of this study should be interpreted in view of the following limitations. First, the design of this study does not allow conclusions on the direction of causality. It remains to be investigated whether the higher positive scores in patients from “at risk for psychiatric disorder” families are indicative of an increased familial loading for psychosis or whether these higher scores lead to higher psychosis dimension scores in the relatives. Second, only a limited number of etiological variables were assessed. So far, genetic variation appears the most important risk factor for schizophrenia, but we do not have the data needed to compare genetic risk between subtypes. Finally, while the advantage of our new approach is the use of more refined measures to define family subtypes, these refined measures are only available in the relatives who participated in the study. Not all relatives were willing to participate and we can not rule out the possibility that willingness to participate is associated with the level of symptomatology.

## Conclusion

We have used a new approach to identify different family subtypes based on the level of subclinical symptoms in the relatives of schizophrenia patients. Our findings confirm the existence of high-risk families and suggest that future studies on the etiological factors of schizophrenia should differentiate between individuals from “at risk” and “healthy families”.

## Competing interests

LdH has received research funding from Eli Lilly and honoraria for educational programs from Eli Lilly, Jansen Cilag, BMS, Astra Zeneca. JvO is/has been an unrestricted research grant holder with or has received financial compensation as an independent symposium speaker from Eli Lilly, BMS, Lundbeck, Organon, Janssen-Cilag, GSK, AstraZeneca, Pfizer and Servier, companies that have an interest in the treatment of psychosis. All remaining authors have declared that there are no conflicts of interest in relation to the subject of this study.

## Author’s contributions

EMD, MPM and JV analyzed the data and wrote the manuscript. RSK, DHL, JvO, DW, RB, WC, LdH, LK, and IM-G made substantial contributions to study design and have been involved in drafting the manuscript. All authors read and approved the final manuscript.

## Pre-publication history

The pre-publication history for this paper can be accessed here:

http://www.biomedcentral.com/1471-244X/12/71/prepub
